# Tartar, or Calculus of the Mouth

**Published:** 1844-06

**Authors:** 


### ARTICLE VIII.

*Tartar, or Calculus of the Mouth.* Translated from the French of DELLABARRE, by * A *

TARTAR, stone, or *odontolithos,* is a calculous substance that collects upon the teeth. Various analyses have been made of it, both by French and foreign chemists; but every analysis given of it, has necessarily differed from another, because the tartar thus analyzed, was taken from subjects of different temperaments, of which circumstance the analyzers appear to have taken no account. This stony substance, however, is far from being of the same nature and containing the same principles in all individuals; thus, for example, the black dry tartar which is found in small quantities around the necks of the teeth of persons having a good constitution, dissolves with difficulty in muriatic acid. The yellow, dry tartar of persons called bilious, dissolves much more readily, while the white, soft tartar of mucous temperaments, scarcely at all soluble in the acids, is readily dissolved in the alkalies. This last contains a great proportion of fibrin, but the others have more of the terreous basis. Exact and comparative analyses of the different sorts of odontolithos, are yet to be made, and I do not doubt that great difference will be found to exist between them. Though chemists should have taken every precaution that could have rendered their labors of utility to the physician, there still would remain a question to be discussed which is infinitely of more importance to the physiologist. It consists in the solution of the following problem :

Whence proceeds the calculus of the mouth ? Is it, as some have said, a secretion ? Is it a deposite, as has been repeated again and again, of the saliva, as stated in every medical work that has appeared for centuries ? or, is it not rather a diseased terreous exhalation of the mucous membranes of the gums ?

Jourdain and some others have supposed, that the small glands scattered over the periosteum of the teeth, secrete the tartar. Gariot simply observes, that it comes from the gums ; but a late author announces that he has discovered some *dental glands*, which should more properly be called *calculars* : as if nature had furnished a system for this noxious formation. But is not this author too hasty in supposing their existence ? The small glands that he thus designates, may perhaps belong to the mucous and salivary systems, for the saliva, as all physiologists know, is not furnished by the parotid glands alone, but also by a great number of calculous kennels, which are very observable in ruminating animals, distributed over different parts of the mucous membrane of the mouth. I therefore think that this is a gratuitous supposition on the part of this author, because children of a very early age, are not affected with tartar, and it is on them that he thinks he has discovered the glands which produce it. Did these really exist, they would instead of decreasing, augment in volume, as age advanced, and their functions being more and more established, they would be very large in old persons and those who are subject to tartar. Now there is nothing to lead us to suppose their existence in these individuals. To suppose, therefore, that organs without functions may be very large, which, when they have them, cannot be discovered, is contrary to sound physiology ; were we to do so in this case, the *dental glands* of the author, would be wholly different from all others, which are the more decided the more they are in action. This supposition being inadmissible, I do not believe in the existence of these glands, which I have patiently searched for, but in vain.

Notwithstanding the opinion of some surgeon dentists-experimentalists, the most accredited hypothesis in the formation of odontolithos, is, that the terreous salts contained in the saliva are precipitated by a chemical agent, and gradually deposited on the teeth, where they are agglutinated by means of the mucus of the mouth.

This supposition is admitted by the English dentist, Fox, and is generally followed without examination by most of our modern elementary works; it is, say they, by analagous means, that the calculi of the bladder, the kidneys, the gall vesicle, &c., are formed;* but has this interesting point of physiology and pathology, been considered in every point of view? Calculi are found not only in cavities designed to serve as reservoirs for certain fluids; but they are also met with in the stomach and intestines of some animals, in the articulations of gouty persons, in the peranchyma of the liver and lungs, in the thickness of the muscles, and finally in the brain itself, where they have been mistaken for osseous portions of its subtance.†

It is, nevertheless, worthy of remark, that the mucous membranes, as well as the sinovial glands and those that furnish the mucus are more especially liable to the production of concretions. Thus they are frequently found in the tonsils or amygdales, in the nose, the maxillary sinus, the external auditory conduit, the articulations, &c., whence it appears to me more natural to suppose them to be the product of an accidental exhalation of the sanguinary capillaries, to which the mucous and sinovial systems are much disposed.

Veterinary physic furnishes us with proofs to the point of what I have advanced. M. Duhuy, professor of the veterinary establishment, of Alfort, has demonstrated that the mucous membranes of certain animals are subject to a disorder that he calls tuberculous, which consists in the formation of a fine gravel that is found in the thickest parts of their tissue, and whose analysis gives almost the same products as the other stones.

Surely these sorts of concretions as well as those that are

* Vide Researches on the causes of Gravel by Magendie, 1818. The Dictionary of Medical Sciences, and an Essay on the Chemical History and Medical Treatment of Calculous Disorders.—London.

† Vide the Anatomy of the Brain, by Dr. Gall.

found either in the nose, or in the maxillary sinus, &c., can only come from a diseased exhalation of the membranes, *since there is not in these places, any other fluid than the mucus; and even it remains there but a short time.*

In the same manner the exhalents of the gums appear to me to furnish tartar; they give out more or less of it, according as the gums are healthy or inflamed; the blood that penetrates their capillaries contains more or less calculous earth. This is a remark, to which I have often called the attention of my pupils that have been present, on my visit to the orphan asylum, or at my private consultations. When the gums are diseased, they are covered with a whitish layer, which is soft at first, but gradually collects upon the teeth, where it hardens. When they are not inflamed they do not produce it, so that we sometimes observe the teeth on one whole side of the mouth, where the gums are very sound, not having a particle of calculus on them, while on the opposite side where inflammation does exist, there is a considerable quantity of it, yet the saliva bathes the whole of this cavity. This observation may easily be made: 1st, in children that are changing their teeth; because on the side where the molting is going on, their gums are more or less irritated. 2d, On a person who habitually has no tartar on his teeth, but who being attacked with a fluxion in only one of his cheeks, is affected with a catarrh in the mucous membrane of the mouth. The side affected presents a viscous saliva or gums covered with slime; whilst on the other side there is nothing of the sort. If we remove the cheek with a spoon, and observe the oozing of the saliva, we shall find, it is true, that it flows in greater abundance than usual, but yet is very limpid. 3d, If we examine the saliva that comes immediately from the parotid, without having entered the mouth, but diffuses itself over the cheek because of a fistula in the conduit of the gland, we shall see it fall drop by drop, without being viscid: if we then collect and analyze a portion of it, we shall find that there is no calcareous phosphate in it, but, if from the same subject, we take the saliva of the mouth, we shall discover that it is more or less mucus, and that the terreous salts are the more abundant the greater the proportion of mucus. 4th, The conduits of stenon were removed from a horse,* in order to obtain

* An experiment made at the Veterinary establishment, at Alfort.

the fluid which is secreted by the glands, and on its being analyzed, no calcareous earth was found. Concretions in the conduits of this animal are frequently found; they consequently must be the product of an exhalation from the mucous membrane that lines the conduits, and not a deposite of the saliva which passes over them.

I have in general observed, that in such a state of health as the subject is usually in, whatever may be his age or temperament, the quantity of calculus, is in direct ratio to the proportion and consistence of the mucus. It is also in an increased ratio to his strength.* Thus for example, the saliva of a good constitution, and in the vigor of youth, has an affinity for atmospheric air, with which it mixes, so as to become frothy; it floats on the top of distilled water, and soon mingles with it; there is little or no tartar on the teeth; but as soon as disease or age has weakened him, his saliva becomes mucus, floats below the surface of distilled water, does not mix with it, and frequently sinks to the bottom. There is also a greater or less quantity of tartar in different parts of the mouth. The principal and quantities of the mucous portion of the saliva vary with the state of health according to age and temperament; hence it happens, that the calculus of the mouth is presented under different aspects; sometimes it appears in great abundance, as a sort of slime; at one time it constitutes a very hard and blackish body, at another, it collects in thick yellow crusts.

The analysis of the odontolithos should consequently give results differing according to physical character. This is the case with other calculi, which always contain materials depending on locality. Thus uric acid is found in those of the bladder, while the fluids of the adipose vesicle, are odorous by reason of the *debris* of vegetable matter which they contain, &c.

I have frequently observed, that when the mucous membrane of the gums, irritated by some local or general cause, has become inflamed, and the resolution has become imperfect, these results are atonic obstruction, the gums continue swollen, and discharge

* I mean by this, the natural strength of each individual accidentally increased or diminished.

with facility, a red purple blood; and, although they, before they were in this state, gave but little tartar, yet now they supply a good deal. Here the formation of the tartar is occasioned by a chronic catarrhal affection of the membrane of the mouth, which must be subdued: 1st, by removing tartar and paying daily care to cleanliness: 2d, by resolutive and sometimes acidulated gargles, and finally by spirituous and astringent tonics; but if these means are not sufficient to restore energy to the vessels of the gums, which are afflicted with a species of debility, the embrocatibility of their tissue is destroyed, the calculous matter spreads over them, the dental periosteum dries up, purulent matter oozes out between the gums and teeth, which causes them to fall out by excavation, although they are perfectly sound.

Accidental or acute inflammation of the gums very easily passes to the chronic state in persons who delay to have it reduced. To effect its reduction, we are sometimes obliged to have recourse to various therapeutic means, and, though we cannot expect to prevent entirely, the formation of tartar, yet we may very much diminish its quantity; to this end, repeated purges and vesications are, when properly employed, infallible means: I have frequently ordered them and have obtained success, even after the fluxions had passed to the state of catarrh. It was, therefore, not without pleasure, that I read in the second article of an analysis, of an essay on the chemical history and medical treatment of calculous diseases by M. Marcet, a physician of London, that he had made the same remark in regard to the vesicular calculi.* Here the purgation acts by removing the seat of irritation.

When we reflect that persons most subject to odontolithos have foul tongues, in the morning before eating, must we not believe that this disposition is generally throughout the whole extent of the intestinal membrane, and that these temperaments are very improperly called bilious? Do not the affections that are peculiar to them rather depend on a disposition of the mucous mem-

* See the Journal edited by M. M. Bechard, Magendie, vol. 1, p. 375.

branes, which more or less abundantly secrete this yellow filth that closely resembles soft tartar, and contains much mucilage?*

From what I have just said, the formation of tartar may depend either on an idiosyncrasy of the gums, a local irritation, a general weakness of the solids, or finally on a superabundance of earth in the fluids. The true cause, therefore, can be known, not by a superficial examination of some parts of the gum, but by a close inspection of every part of the mouth. The tartar that is the result of local inflammation, generally encrusts, only on a few of the teeth, it is hard, and the gums are sound in every part where it is not present; it is found on teeth that become painful in consequence of inflammation in the periosteum. That which depends on an idiosyncrasy is spread over the whole mouth, and may be of considerable consistence; that which is deposited in consequence of general weakness of the vessels is small in quantity, white or yellow in color, and thickly impregnated with mucus. The gum is swollen, soft, and slimy, such as we observe in persons attacked with scorbutus. That which results from a superabundance of earth in the humors, is frequently dry and hard, it concretes upon all the teeth, and is thickly packed upon the gums, causing them to swell but not depriving them of their firmness. This last sort is found on aged persons and those of a bilious temperament; in the greater number of instances, the tongue is continually covered with a foul yellowish coat.

Men are not alike subject to calculus of the mouth, in all climates of the globe. In general, the travellers that I have consulted on this subject, have assured me, that in Asia, Africa, and in all those regions, where the heat is great, the teeth are good and but little affected with tartar. The same is the case in elevated and temperate countries; but in places that are cold, marshy and subject to fogs, the mouth easily contracts a catarrhal affection, which produces much filth. But without extending our

* We may be assured of the truth of what I have advanced, by a very simple experiment, which consists in collecting the filth for a fortnight, with a tongue scraper, and placing it in a glass of water; we must decant the water every morning and pour in fresh. It will then form at the bottom of the vessel a yellow or greasy precipitate, having all the characteristics of tartar.

observations so far, do we not find, that the gay inhabitants of Provence, generally need but little assistance from the dentist, while in the western parts of France, such as Havre, Dieppe, &c. there are multitudes whose teeth are bad and thickly encrusted with tartar.

As it regards age, children generally have but little tartar, unless they are in a bad state of health.

Adults have more of it, especially after having passed their thirtieth year, and advance towards old age.

It is in the last periods of life, that the mouth presents the largest quantity of it, because then, the calcareous earth, that abounds in our humors, becoming superfluous, exudes by means of the exhalents from the mucous membranes and the skin.

As to temperament, persons of a sanguinous temperament have but a small quantity of tartar, those of mucous, much; those of a bilious, more; and its chemical and physical characteristics vary in each.

The relative state of health in each temperament also causes the proportion of tartar to vary; and the physician will profit by the remark; for the more abundant formation of tartar, where there is no local inflammation capable of determining it, is a consequence of a weakness in the solids of the subject; whence a change in the qualities of the fluids results.

If we turn our attention to the bladder, in order to study by analogy the formation of stones, we shall be led to apply the same principles; when we remove a sound that has remained for some length of time in this cavity, we frequently find it covered with a calculous incrustation at different points, although the patient has never experienced any thing announcing a disposition to gravel. In my medical practice, I have had frequent opportunities to observe such cases, and a very remarkable one is recorded in the Journal of *Sidillot* for July, 1818, which gives an account of a soldier in good health, in whom a sound was employed, to prevent the obstruction of the urinary canal. Having been neglected, it remained in the bladder for seventy-nine days; at the end of which time it was resolved that the operation of lithotomy should be performed, in order to remove a calculus that was attached to it, and which prevented it from being withdrawn, but the patient fearing the bistoury, had the sound pulled out by main force. It was found to be covered with thickened mucus, which had entirely blocked it up; it was corrupted on its surface, rough for the distance of two inches, and bore on the extremity that had been in the bladder, a urinary concretion, that was rough in its appearance, of an oval form, and about the thickness of an almond. It appears to me to be evident that, in this case, the presence of the sound determined on the parts of the membrane that it touched, a slight irritation, the result of which was an exhalation of a concrescible substance, for had that sort of petrifaction been contained in the urine, properly so called, it is certain that all parts of the sound which had been in the bladder, would have been alike covered with it.

The analysis of the products of the mucous membranes, shows that there is a great proportion of terreous salts in them; it is not, therefore, erroneous to suppose that the source of these salines terrene animal concretions, is in the capillary system of these vessels. Would it not be possible to take nature in the act, by analyzing first the urine of an animal that had never before been subjected to any experiment, and then removing one of the uriters, in order to collect the urine that comes from the kidney alone, and make the same analysis of it? By the means that I propose, we should be able to appreciate the additions which this liquid receives while remaining in the bladder, and to discover the qualities of the terreous salts that are deposited by the mucous membrane of this cavity, just as we find that the saliva when it comes from the glands is limpid and soapy, but by mixing with the mucus of the mouth becomes foul, stringy, and sometimes acidulated. Thus, therefore, as I believe it to be demonstrated, that *tartar is furnished by the mucous membrane of the mouth*, so also, I think that the calculi of the kidneys, and the bladder, as well as the very abundant sediment, by which the presence of certain diseases is conjectured, come from the urinary mucous membrane. This opinion may give rise to some considerations, into which, I cannot in this work, enter. For example, the gout more especially affects the articulations, and frequently determines inflammation there. I have from curiosity dissected several persons who had been but for a short time afflicted with the gout, and I found their sinovials were only inflamed, but when the disease has been of long continuance, there is a deposite of a calcareous substance. The capillaries of these membranes, therefore, deposite tophacious materials, which are consequently the effect, and not the cause, of chronic inflammation, of which the articulations of gouty persons are the seat.

Again, the gall does not contain any adipose, which, however, is found in the calculi of the vesicle, consequently the mucous membrane must secrete this substance, which protects it from the irritating action of the gall, as it also does, that which comes from the exhalation of the cerum of the ears, where also we meet with petrifactions. In birds, the material for the shell of the egg, is furnished by a peculiar secretion of the intestine membrane.

In ruminating animals, a small piece of wood that has not been digested in the stomach, and whose stay there is often prolonged, determines a slow irritation that is followed by a salino-terreous exhalation, which by accumulating and fastening itself to this foreign body gives rise to the formation of calculi. Finally is it not manifest, that the small calculous masses that are found in the stomach of the craw-fish, are furnished by the membrane which lines this cavity?

The mode in which these concretions are formed in other animals, has led me to these various reflections on those that are met with in different parts of the human body, especially the bladder. Indeed, we frequently find some foreign body at their centre, which has been accidentally introduced into this cavity by the urinary canals; now a small pebble, for example, cannot change the nature of the urine, or increase the natural quantity of the uric acid, &c., but it irritates the urinary mucous membrane, which becomes inflamed, contracts a catarrhal affection and exhales terreous salts—the base of the calculus enveloping the foreign body.

If we operate on the patient, the disease is rarely reproduced, because the cause is no longer existing, the irritation of the mucous membrane ceases, and the operation otherwise changes the mode of sensibility of the bladder; but if the catarrh has passed to a habit, there may be a relapse.

Were the kidneys destined by nature not only to secrete the urine, but also to separate from the blood the terreous salts which are discovered when an analysis is made of the urine that comes from the bladder, the operation of lithotomy would only be a means of affording momentary relief to the patient, but could not radically cure the disease, for, scarcely would the calculus be removed, and the wound cicatrised, before the urine would deposite anew the calculous matter, so that it would be necessary either to operate once a year, or every six months, or to keep the patient under a severe and slightly nutritive regimen, to which few would submit, and which, indeed, they could not endure.

It appears to me, therefore, that all the various petrifactions that are met with in so many different tissues of the economy, indicate a mode of formation independent of all prior suspension of calcareous earth in a fluid, and subsequent precipitation thence, by a chemical action. I accordingly suppose, that the saline materials contained in the article blood, penetrate, in consequence of a chronic local affection, into the capillaries of the different systems, whose exhalent mouths allow them to exude.

I will here terminate these observations by remarking that the opinion which I have for a long time entertained in relation to the mode of formation of calculi in general, appears to me to be very like that of Hunter's, which may perhaps give it some weight. This celebrated surgeon also intends to compose a special work on this subject, but if he has ever published such a one, I have never been able to procure it.

Although the ideas that I have just thrown out, have their origin in my intimate conviction of their truth, as regards the calculus of the mouth, and although they differ from those of physiologists, after whom M. Magendie, in France, and M. Marcet, in England, have published two very good treatises, the former, on gravel, and the latter, on visicular calculi. I would, nevertheless, observe, that the means which they point out to prevent or cure calculous diseases, would also be those to which we should be obliged to resort, to prevent the formation of tartar, did we not possess the advantage of being able to apply, to the mucous membrane of the mouth, the local remedies proper to oppose its accumulation. Finally, everv regimen tending to diminish the quantity of terreous salts that enter into the composition of our bodies, will be a preservative from odontolithos, as well as from the other calculi.

